# The Role of Thyroid Disorders, Obesity, Diabetes Mellitus and Estrogen Exposure as Potential Modifiers for Pulmonary Hypertension

**DOI:** 10.3390/jcm11040921

**Published:** 2022-02-10

**Authors:** Eleni Vrigkou, Evangeline Vassilatou, Effrosyni Dima, David Langleben, Anastasia Kotanidou, Marinella Tzanela

**Affiliations:** 1 1st Department of Critical Care and Pulmonary Services, School of Medicine, National and Kapodistrian University of Athens, Evangelismos Hospital, 10676 Athens, Greece; elenivrigkou@gmail.com (E.V.); efi_dima@yahoo.gr (E.D.); akotanid@med.uoa.gr (A.K.); 2 Endocrine Unit, Hygeia Hospital, 15123 Athens, Greece; evassilatou@gmail.com; 3 Center for Pulmonary Vascular Disease, Azrieli Heart Center, Jewish General Hospital and McGill University, Montreal, QC H3A 0G4, Canada; david.langleben@mcgill.ca; 4 Department of Endocrinology, Diabetes Center, Evangelismos Hospital, 10676 Athens, Greece

**Keywords:** diabetes mellitus, estrogen, obesity, pulmonary hypertension, thyroid disorders

## Abstract

Pulmonary hypertension (PH) is a progressive disorder characterized by a chronic in-crease in pulmonary arterial pressure, frequently resulting in right-sided heart failure and potentially death. Co-existing medical conditions are important factors in PH, since they not only result in the genesis of the disorder, but may also contribute to its progression. Various studies have assessed the impact of thyroid disorders and other endocrine conditions (namely estrogen exposure, obesity, and diabetes mellitus) on the progression of PH. The complex interactions that hormones may have with the cardiovascular system and pulmonary vascular bed can create several pathogenetic routes that could explain the effects of endocrine disorders on PH development and evolution. The aim of this review is to summarize current knowledge on the role of concomitant thyroid disorders, obesity, diabetes mellitus, and estrogen exposure as potential modifiers for PH, and especially for pulmonary arterial hypertension, and to discuss possible pathogenetic routes linking them with PH. This information could be valuable for practicing clinicians so as to better evaluate and/or treat concomitant endocrine conditions in the PH population.

## 1. Introduction

Pulmonary hypertension (PH) is a progressive disorder characterized by a chronic increase in pulmonary arterial pressure, frequently resulting in right-sided heart failure and potentially death [1]. According to the latest European Society of Cardiology/European Respiratory Society (ESC/ERS) guidelines published in 2016 [1], PH has been traditionally defined as a mean pulmonary arterial pressure (mPAP) ≥25 mm Hg at rest measured by right-heart catheterization (RHC). In the Sixth World Symposium on PH in 2018, however, it was suggested that PH should be diagnosed when mean pulmonary arterial pressure is greater than 20 mm Hg measured by RHC at rest [2]. PH is categorized into five groups based on clinical presentation, and pathological and pathophysiological findings: (1) pulmonary arterial hypertension (PAH), (2) PH due to left-heart disease, (3) PH due to lung diseases and/or hypoxia, (4) PH due to pulmonary artery obstruction (e.g., chronic thromboembolic PH (CTEPH)), and (5) PH with unclear and/or multifactorial mechanisms [3]. PAH is a distinct PH group whose pathobiology arises mainly from the pulmonary vascular bed; it is defined, as per the ESC/ERS guidelines, by an elevated mPAP (≥25 mm Hg) and pulmonary vascular resistance-PVR (>3 Wood Units), combined with a pulmonary arterial wedge pressure ≤15 mm Hg, at rest by means of RHC [1].; of note that mPAP >20 mm Hg and PVR ≥3 Wood Units are the proposed changes in the Sixth World Symposium [2]. PAH can be idiopathic, heritable, or associated with various medical conditions (e.g., drug and toxin exposure, connective tissue diseases, portal hypertension, congenital heart diseases) [1,3]. In this review, we will use the term PAH or the ones of other abovementioned PH groups [3], when specified in the cited references. When the latter is not met, the general term PH will be used. 

PH is an umbrella hemodynamic term that includes around 30 clinical conditions that can lead to pulmonary arterial pressure elevation [4]. Co-existing medical conditions are important factors in the generalized PH setting, since they may not only result in the genesis of PH, but may also contribute to the progression of the disorder [5]. Examples of such medical conditions are thyroid disorders [5]. Thyroid disorders are listed in the ESC/ERS guidelines among the etiological factors for the development of Group 5 PH (PH with unclear and/or multifactorial mechanisms) [1]. They have been associated, however, with other forms of PH as well; observational data demonstrated a prevalence of thyroid dysfunction in other than Group 5 PH patients, as stated by the authors [6]. Several studies also suggested that immune thyroid diseases are commonly found in the PAH population [7]. 

In the last 40 years, various studies have been conducted with the purpose of assessing the relationship between endocrinopathies and PH [8]. Thyroid disorders are amongst the most studied endocrine diseases in the PH setting [9]. Other endocrine disorders (namely estrogen exposure, obesity, and diabetes mellitus) have also been evaluated regarding their possible impact on the evolution of PH [8], and PAH in particular [10]. The aim of the present review is to summarize current knowledge on the role of thyroid diseases, obesity, diabetes mellitus, and estrogen exposure as potential modifiers for PH, with a special focus on PAH, and to discuss the possible pathogenetic routes linking these endocrine conditions with PH.

## 2. Thyroid Dysfunction and PH

### 2.1. Thyroid Hormones

The thyroid gland is the only source of 3,5,3′,5′tetraiodothyronine or thyroxine (T4), while it secretes approximately 20% of the daily produced 3,5,3′triiodothyronine (T3), as most of this hormone (~80%) is derived from peripheral tissue (mainly hepatic and renal) deiodination of T4. Thyroid hormones circulate in blood either unbound (free form) or bound to plasma proteins, with thyroid hormone binding globulin (TBG) being the main transport protein. Free thyroid hormones, which are available for entry and action in target tissues, circulate in extremely small amounts as they represent approximately 0.04% of total circulating T4 and 0.4% of total circulating T3. 

Thyroid hormones have direct effects on the cardiovascular system, since thyroid hormone receptors are located in the myocardial and vascular endothelial tissues [11]. Moreover, they can produce indirect effects on cardiac and vascular structures via the adrenergic nervous system [12]. Evidence in the literature suggests that the cardiac and vascular alterations caused by thyroid hormone abnormalities could have as an end-target the pulmonary vascular bed inducing a hypertensive condition [10].

### 2.2. Thyroid Diseases in PH Patients

Studies have shown that thyroid diseases are more prevalent in PH in general [9,13], and PAH in particular [7], compared to age- and sex-matched healthy controls. Li et al. reported that the prevalence of thyroid diseases was also elevated in PH patients when compared to patients with pulmonary diseases [14]. In the PH population, the presence of thyroid disorders has been estimated by some reports to be up to 24% with hypothyroidism being the predominant thyroid disease [14]. Moreover, among the PH subgroups, the one with the highest prevalence of thyroid conditions was PAH (and especially idiopathic PAH) [6,15]. The Registry to Evaluate Early and Long-term PAH Disease Management (REVEAL) and related studies have confirmed the above-mentioned data by reporting that comorbid thyroid conditions are commonly found in PAH patients, either present at diagnosis or developing during the course of the disorder [7,10]. Data on the prevalence of PAH in patients with hypothyroidism are limited to case reports [16]).

Thyroid dysfunction and thyroid replacement therapy have also been identified as risk factors for the development of Group 4 PH, i.e., CTEPH [17,18]. Hypothyroidism was found as the predominant thyroid condition and was associated with more severe cases of CTEPH [19]. Robledo et al. reported that PAH patients without hypothyroidism had better functional capacity, pulmonary hemodynamics, and walked greater distances in the six-minute walk test compared to PAH patients with hypothyroidism [20]. Vakilian et al. reported that almost half of their idiopathic PAH patient cohort presented with subclinical hypothyroidism [21]. They also associated the concomitant hypothyroidism with lower functional capacity and poorer patient outcomes [21].

Other studies have associated low free T3 levels in PAH and CTEPH patients with increased mortality [22]. Richter et al. reported that idiopathic PAH patients with high and low thyroid-stimulating hormone (TSH) values presented increased risk of life-threatening events [22]. Despite these data, the available evidence in the international literature is still not strong enough to support a causative relationship between TSH, T3, and T4 levels or treatments for thyroid disorders and patient outcomes [21]. In the Li et al. study, 59% of the PH patients (consisting of patients belonging in PH Groups 1, 2, 3, and 4) with concomitant hypothyroidism received adequate thyroid treatment and had normal TSH values [14]. In this study, thyroid treatment and TSH values were not linked to clinical outcomes since it was out of the scope of this research; the authors reported, however, that the correction of thyroid abnormalities did not seem to influence the severity of the disorder.

Autoimmune thyroid diseases (AITD) have been implicated as potential modifiers for PAH: Chu et al. reported that almost half of the PAH patients in their cohort were diagnosed with AITD (Hashimoto’s thyroiditis, Graves’ disease or euthyroidism in the presence of antithyroid antibodies) [23]. Another study has shown that children and adolescents with idiopathic PAH also presented with a high prevalence of AITD (Graves’ disease, silent thyroiditis, or antithyroid antibodies with euthyroidism) [24]. It should be noted that the term “silent thyroiditis” is used in the latter reference, although this diagnosis is usually a variant of Hashimoto’s disease. The authors defined “silent thyroiditis” as the combination of findings of primary hyperthyroidism, a diffuse goiter without pain, and normal blood flow in the thyroid gland on ultrasonography, whether or not TRAb (thyroid-stimulating hormone receptor antibody) was present [24].

Numerous studies and case reports have linked hyperthyroidism with PH [25,26,27], and in particular with PAH [28]. Most reports on the subject describe mainly new-onset PH (often severe) in patients with hyperthyroidism and the predominant thyroid abnormalities found are Graves’ disease and multinodular goiter-induced hyperthyroidism [26]. This may be the result of direct actions of thyroid dysfunction on the pulmonary circulation but, in many other cases, the pulmonary hypertension may result from hyperdynamic high cardiac output and left heart dysfunction, resulting in postcapillary PH. In the pediatric population, a link has also been shown between PAH and hyperthyroidism [29]. The studies conducted in the adult and pediatric populations suggest that appropriate treatment of hyperthyroidism can result in the improvement or resolution of PH [29,30,31]. Specifically in pediatric patients, Trapp et al. reported that aggressive treatment of hyperthyroidism in children with PAH could reduce the likelihood of life-threating events [29].

Apart from the effect that treatments for thyroid disorders may have on the progression of PH, evidence suggests that PH-specific treatments can also affect PH patients’ thyroid status. Initiation of medications affecting the prostanoid pathway has been associated with the development of thyroid conditions in PH patients, mainly thyrotoxicosis and goiter formation [32,33]. It has been suggested that this could be attributed to the direct effects that prostanoids could have on thyroid tissue and thyroid function [32,33], but may also just be the coincidence of starting therapy in PH patients who would have developed thyroid disease anyway.

### 2.3. Biologically Plausible Pathogenetic Mechanisms Linking Thyroid Disorders with PH

Despite literature data highlighting the potential relationship between PH and thyroid dysfunction, the underlying mechanisms explaining this relationship are poorly understood [6]. Several authors, however, have proposed potential pathogenic routes that could explain how thyroid dysfunction may affect the progression of PH. 

Hypothyroidism: In hypothyroidism, the low levels of thyroid hormones could have direct effects on the cardiovascular system, leading to decreased cardiac contractility, elevated diastolic blood pressure, and increased systemic vascular resistance [11]. These alterations could result in the reduction of cardiac output, the elevation of pulmonary arterial pressure, and, potentially, in the proliferation of the pulmonary vascular walls [34,35]. Thyroid hormones bind on thyroid hormone receptors located in vascular smooth muscle cells signaling the phosphatidylinositol 3-kinase/protein kinase B (PI3K/PKB) pathway [36]. Low levels of T3 and T4 could affect the PI3K/PKB pathway resulting in decreased expression of nitric oxide synthase isoforms in endothelial and muscular cells, and consequently in the decreased release of nitric oxide, decreased vasodilation and endothelial dysfunction of the pulmonary vasculature [37]. Hypothyroidism can also interfere with the production of adenosine (via the ecto-50-nucleotidase enzyme) and, thus, impair the adenosine-mediated vascular relaxation process [38]. Low levels of thyroid hormones have also been suggested to promote vascular remodeling, by interfering with the cyclic adenosine monophosphate response element [15,39].

Several authors have suggested that autoimmunity may play a decisive role in the development of both thyroid disorders and PAH [34]. It has been hypothesized that the underlying systemic vascular inflammation caused by autoimmunity can promote angio- proliferation and vascular remodeling of the pulmonary bed, contributing to the development of PAH [40].

**Figure 1:**
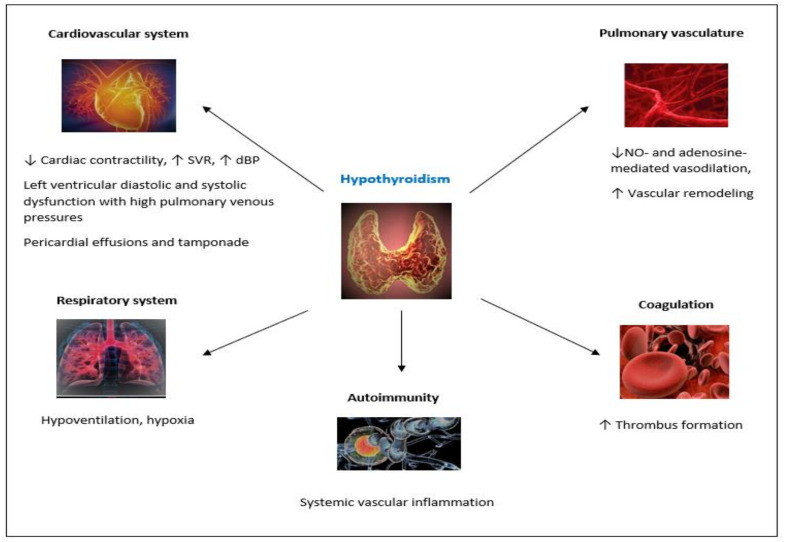
Biologically plausible pathogenetic routes (indicated by arrows) linking hypothyroidism with PH. Abbreviations: dBP, diastolic blood pressure; NO, nitric oxide; SVR, systemic vascular resistance.

Hypothyroidism is associated with hypoventilation and hypoxia [16]. Hypoxic conditions can induce vasoconstriction of the pulmonary vasculature and, consequently, worsen the co-existing PH [11]. Some studies have also linked hypothyroidism to coagulation disorders, which can lead to increased thrombosis generation and subclinical embolization of the pulmonary bed [41]. Lastly, thyroid diseases have been associated with mutations in the gene encoding the bone morphogenetic protein receptor type II (BMPR2), a well described genetic cause of PAH [42]. The proposed pathogenetic routes linking thyroid dysfunction with PH are summarized in Figure 1.

Hyperthyroidism: Hyperthyroidism may have various effects on the cardiovascular system. The elevated levels of thyroid hormones can have positive inotropic and chronotropic actions on the myocardium [11]. They can also lead to the decrease of peripheral vascular resistance and diastolic arterial pressure, resulting in the activation of the renin-angiotensin-aldosterone system and, consequently, in the increase of blood volume [12]. These cardiovascular effects may result in the elevation of cardiac output, which in turn could lead to hyperdynamic pulmonary circulation and high cardiac output-induced endothelial injury [34]. More importantly, and likely the most frequent cause of pulmonary arterial pressure elevation in patients with hyperthyroidism is high-output left heart failure. In this case, the elevation of left-heart filling pressures is transmitted backwards into the pulmonary vasculature, resulting in group 2 PH (PH due to left-heart disease).

Several authors have hypothesized that autoimmunity may play an important role in the development and evolution of PH in patients with hyperthyroidism. It has been suggested that an autoimmune mechanism could be associated with vascular endothelial dysregulation or damage [43,44]. To date, the exact immune- mediated process that explains how hyperthyroidism affects the pulmonary vasculature has not been well-defined [22]. Nicolls et al. suggested that immune-mediated injury of the endothelium could lead to endothelial cell destruction, pulmonary vascular remodeling, and, consequently, pulmonary arterial pressure elevation [45]. Sugiura et al. hypothesized that autoimmune-mediated pulmonary vascular remodeling may be an important factor in Graves’ disease-linked PH [43].

**Figure 2:**
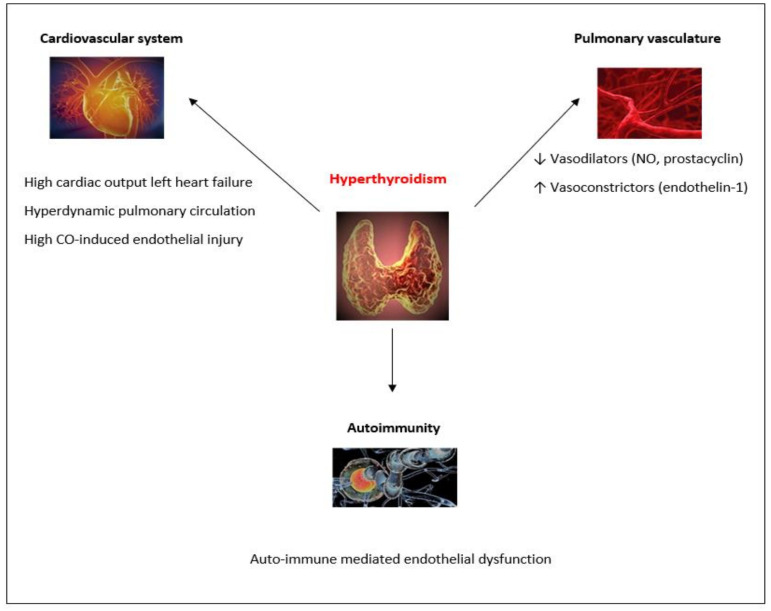
Biologically plausible pathogenetic routes (indicated by arrows) linking hyperthyroidism with PH. Abbreviations: CO, cardiac output; NO, nitric oxide.

Studies have shown that hyperthyroidism can interfere with the production of pulmonary vasodilators and vasoconstrictors. More specifically, hyperthyroidism can decrease the production of intrinsic pulmonary vasodilating substances (including nitric oxide and prostacyclin) and increase vasoconstrictors (like endothelin 1 and thromboxane) [12]. These alterations can result in the elevation of pulmonary vascular resistance [11]. Lastly, other proposed pathogenetic mechanisms explaining the relationship between hyperthyroidism and PH include enhanced catecholamine sensitivity, alterations of the energy metabolism and thyroid hormone- induced angioproliferation [8]. Figure 2 summarizes the above-mentioned pathogenetic routes.

In summary, observational data (although limited) suggest that thyroid diseases are common in PH patients and may be associated with adverse outcomes. Even though there are no guidelines so far on the management of thyroid conditions in PH patients, known thyroid disorders should be treated adequately and screening for thyroid diseases in PH patients with no known thyroid abnormalities is a practical approach. In particular, this approach is more appropriate for PH patients before the initiation of medications targeting the prostacyclin pathway and during this treatment, given the effects of this class of drugs on the thyroid gland.

## 3. Obesity

Several studies have found a link between obesity and pulmonary arterial pressure elevation [46,47]. Frank et al. showed that the prevalence of PH increased by 28% in individuals with class 3 obesity (body mass index ≥ 40) compared to nonobese individuals [48]. In the PAH setting, the prevalence of obesity has been estimated to be around 30–40% [49]. In a lot of these cases, however, pulmonary arterial pressure elevation may not be really attributed to PAH; obese patients may more likely have occult left heart diastolic dysfunction and/or sleep-disordered breathing.

Obesity has been associated with several conditions, like obstructive sleep apnea, obesity, hypoventilation syndrome, use of anorexigenic medications, cardiomyopathy of obesity, and chronic thromboembolic disease, all of which can affect the development and evolution of PH [50]. Increased body weight can also enhance the systemic vascular inflammation, which in turn can promote angioproliferation of the pulmonary vascular bed and, thus, lead to pulmonary arterial pressure elevation [51,52]. Obesity could result in altered vascular homeostasis (e.g., via changes in insulin sensitivity and secretion, adipokine secretion, oxidative stress, increased pro-inflammatory and decreased anti-inflammatory cytokines) that may predispose obese individuals to the emergence of pulmonary vascular disorders [53].

Studies conducted in order to assess the impact of obesity on mortality in PH have produced conflicting results. Some reports have shown that obesity has no effect on mortality, while others have indicated a protective effect—a phenomenon called “obesity paradox” [48]. This phenomenon could be (at least partly) explained by the fact that the real cause of the pulmonary arterial pressure elevation in some of these patients could be attributed to left heart disease or sleep apnea, which carry better prognosis. Zafrir et al. linked obesity to lower mortality in both pre-capillary and post-capillary PH patients [54]. Weatherald et al. did not report an association between obesity and PAH mortality, however, they did find increased mortality among morbidly obese young patients [49]. McLean et al. found no survival benefit from obesity in PAH patients [55]. Despite the conflicting results, all authors agree that these data should be interpreted with caution and that further research is needed in order to fully elucidate the impact of obesity on PH mortality [48,49]. It is also essential for future studies to search meticulously for left ventricular diastolic dysfunction and sleep disordered breathing, before labelling patients as having PAH. 

## 4. Diabetes Mellitus

Diabetes mellitus type 2 (type 2 DM) has been identified as an independent predictor for developing PH, even after other factors (e.g., coronary artery disease, congestive heart failure, hypertension, smoking) were controlled for [56,57]. PH patients with type 2 DM were found to have higher mortality rates than those without type 2 DM [57,58]. In the PAH population, glucose intolerance and insulin resistance have been implicated in the development and progression of the disease [59]. PAH- type 2 DM patients were also shown to have significantly lower ten-year survival rates than PAH patients without type 2 DM [60]. Regarding type 1 DM and PH [61], and more specifically PAH [62,63], only a few case reports have been published in the context of autoimmune polyglandular syndrome type 2 and 3.

It has been hypothesized that type 2 DM could affect the evolution of PH because it can lead to right ventricular dysfunction and remodeling [58]. The prognosis of PH is dependable on the patients’ right ventricular ability to tolerate the increased afterload created by PH [1]. Right-ventricular dysfunction induced by type 2 DM, therefore, could affect PH patients’ clinical presentation and the disease’s progression. Furthermore, insulin resistance has been shown to impair endothelial function [64]. More specifically, in type 2 DM vasoconstrictive agents (such as endothelin-1) could be activated, whereas vasodilators (such as nitric oxide) could be decreased [57]. Another important factor to be considered is the high prevalence of occult left ventricular diastolic dysfunction and coronary microvascular disease in diabetic patients. Despite the intense research on the subject, the exact cellular and molecular effects of type 2 DM on the pulmonary vascular bed have not been fully understood [58].

## 5. Estrogen Exposure

The effects that estrogen may have on PAH are, also, a subject of debate in the international literature. On one hand, the female sex is one of the strongest epidemiologic risk factors for PAH [65]. Idiopathic PAH is more common in women than in men, leading to the concept that estrogens may increase susceptibility to PAH. In addition, Sweeney et al. showed that 80% of the female PAH patients reported prior use of exogenous estrogen therapy, either in the form of oral contraceptive pills (premenopausal women) or as hormone replacement therapy (postmenopausal women) [52]. On the other hand, female PAH patients demonstrate superior right heart function and survival rates than their male counterparts [66]. Additionally, several studies using animal models have suggested that estrogens may have a protective role in the PAH setting; however, we must be cautious when extrapolating these findings to humans [66]. This controversy has been often referred to as the “estrogen paradox”.

It is still not clear how sex may contribute to PAH susceptibility. Studies have suggested that female sex hormones may have cardioprotective effects. Estrogen could promote cardiopulmonary neovascularization [67]. They can also inhibit myocardial fibrosis and right-heart hypertrophy, thus exhibiting beneficial effects in PAH [67]. The worse mortality rates and right heart function of the male PAH patients could be explained by the fact that sex hormones might influence the adaptation of the right ventricle to the increased afterload differently in males than in females [66]. On the other hand, the detrimental effects of estrogen could be attributed to data showing that estrogen reduces wild-type BMPR2 expression in pulmonary artery smooth muscle cells and lymphocytes [68].

Estrogens and estrogen metabolites could have diverse effects on the pulmonary vascular bed and this could, at least partly, explain the opposite actions of estrogen presented in the literature [69]. Estradiol for example has been found to promote cell proliferation and pulmonary vascular remodeling [70]. Oxygen concentrations, however, could affect the vascular remodeling actions of estradiol: estradiol could inhibit hyperplasia during hypoxia and promote cell proliferation under normal oxygen levels [71]. Estrogen metabolites may also affect the vascular bed in various ways: 16α-hydroxyestrone has shown to induce proliferation, whereas 2-methoxyestradiol has been found to significantly reduce angiogenesis and remodeling [69,72].

It has been demonstrated that exogenous estrogen can rapidly decrease pulmonary vascular reactivity and acute hypoxic pulmonary vasoconstriction [73]. Estradiol can reduce pulmonary vasoconstriction by promoting nitric oxide production [74]. Since inflammation and autoimmunity play an important role in the PH setting in general, the anti-inflammatory activity of estradiol could also contribute to its beneficial effects [75].

Currently, several therapies targeting estrogen production and signaling are being tested in PAH patients so as to assess their efficacy in treating the disorder [76]. Among the compounds assessed are fulvestrant (an estrogen receptor antagonist), tamoxifen (a selective estrogen receptor modulator) and anastrazole (an aromatase inhibitor) [76]. Kawut et al. performed a randomized, double-blind, placebo-controlled trial evaluating the safety and efficacy of anastrozole in PAH [77]. The authors concluded that anastrazole was well tolerated by PAH patients, who also presented an improvement in a six-minute-walking distance [77].

Pregnancy: Because pregnancy may aggravate PH, or trigger it in a patient with a predisposition, and because it carries a high mortality, current guidelines recommend that PAH patients avoid pregnancy (Class I Level C) [1]. Some case series have described successful outcomes in PAH patients that have responded very well to PAH treatment, but the chance of success is unpredictable, and pregnancy should still be discouraged [1]. In pregnancy, the levels of progesterone, estrogen, dehydroepiandrosterone, and testosterone elevate, leading to vasodilation so as to facilitate the increased plasma volume [78]. Moreover, estradiol and dehydroepiandrosterone have been shown to have beneficial effects on right-ventricular function [78]. Nonetheless, the hemodynamic and volume changes in pregnant PH females frequently increase right-ventricular pressure, which can lead to right-ventricular failure and eventually death [79]. More specifically, the cardiac output demands on an already stressed right ventricle can overwhelm it. Peripartum fluid shifts can lead to loss of right ventricular preload or increased right ventricular filling, resulting in increased wall stress. There is a recognized mortality throughout the later trimesters, but successful delivery does not guarantee maternal survival and early postpartum weeks still carry significant mortality. In patients with other risk factors, such as genetic predisposition, pregnancy may accelerate the pulmonary vascular disease through increased vascular shear stress and it can provoke the appearance of PAH [79].

## 6. Other Endocrine Disorders and PH

The scope of this review focused on thyroid disorders, obesity, diabetes mellitus, and estrogen exposure, as there is increasing evidence on the role of these endocrine disorders in PH development and progression. A short overview focusing on other endocrine pathologies is provided here: A search of the related literature revealed limited data on the association of PH with other endocrine diseases, mainly in the context of secondary hyperparathyroidism in patients with chronic kidney disease and PH [80,81], and of autoimmune Addison’s disease in the setting of autoimmune polyglandular syndrome and PH [61,82].

## 7. Conclusions

PH, and more particularly PAH, are devastating disorders carrying high morbidity and mortality. Research in the field is very active; a new PH definition has been proposed, while new pathobiological findings and therapies are added [83]. There is abundant evidence in the literature suggesting that thyroid disorders, obesity, DM, and estrogen exposure probably act as modulators in the PH setting. Despite the ongoing research, the underlying pathogenetic mechanisms have not been completely clarified. This can be attributed to the complex interactions hormones may have with the cardiovascular system and the pulmonary vascular bed. Confounding effects on the left heart and on breathing must also be considered. Further research is needed in order to elucidate the pathophysiological background linking endocrine conditions with PH. This information is of value to practicing clinicians so as to better evaluate and/or treat concomitant endocrine conditions in the PH patient population. 

## Data Availability

Not applicable.
